# Failure of Translation Initiation of the Next Gene Decouples Transcription at Intercistronic Sites and the Resultant mRNA Generation

**DOI:** 10.1128/mbio.01287-22

**Published:** 2022-06-13

**Authors:** Heung Jin Jeon, Monford Paul Abishek N, Yonho Lee, Heon M. Lim

**Affiliations:** a Department of Biological Sciences, College of Biological Sciences and Biotechnology, Chungnam National University, Daejeon, Republic of Korea; b Infection Control Convergence Research Center, Chungnam National University College of Medicine, Daejeon, Republic of Korea; University of Utah

**Keywords:** transcription pausing, transcription-translation coupling, Rho-dependent transcription termination, cistron junction, *gal* operon

## Abstract

In Escherichia coli, transcription is coupled with translation. The polar *gal* operon is transcribed *galE-galT-galK-galM*; however, about 10% of transcription terminates at the end of *galE* because of Rho-dependent termination (RDT). When *galE* translation is complete, *galT* translation should begin immediately. It is unclear whether RDT at the end of *galE* is due to decoupling of translation termination and transcription at the cistron junction. In this study, we show that RDT at the *galE/galT* cistron junction is linked to the failure of translation initiation at the start of *galT*, rather than translation termination at the end of *galE*. We also show that transcription pauses 130 nucleotides downstream from the site of *galE* translation termination, and this pause is required for RDT.

## INTRODUCTION

The hallmark of bacterial gene regulation is that a group of genes within an operon can be regulated in a coordinated manner by a single process of transcription initiation and termination. In a previous study, we found that 665 of 805 operons of interest use the Rho protein to terminate transcription at the end of the operon (1). To terminate transcription, Rho needs at least 70 nucleotides of the ribosome-free transcript to which it can bind (2–4). At the end of the *gal* operon in Escherichia coli, 73 nucleotides of mRNA downstream from the stop codon of *galM*, the last cistron in the operon, are transcribed to enable Rho-dependent termination (RDT) of transcription. Rho exhibits a high binding affinity for this 73-nucleotide area, which harbors a “C-rich” region with many cytosine residues and few guanine residues (1, 4–6). Rho can bind to the ribosome-free region of the transcript and terminate transcription that has paused at the end of the C-rich region (1). Given that transcription reaching the end of the operon is coupled with translation (7–9), translation termination at the *galM* stop codon decouples transcription from translation, allowing Rho to bind the ribosome-free C-rich region and transcription to end via RDT (1, 10–13).

**FIG 1:**
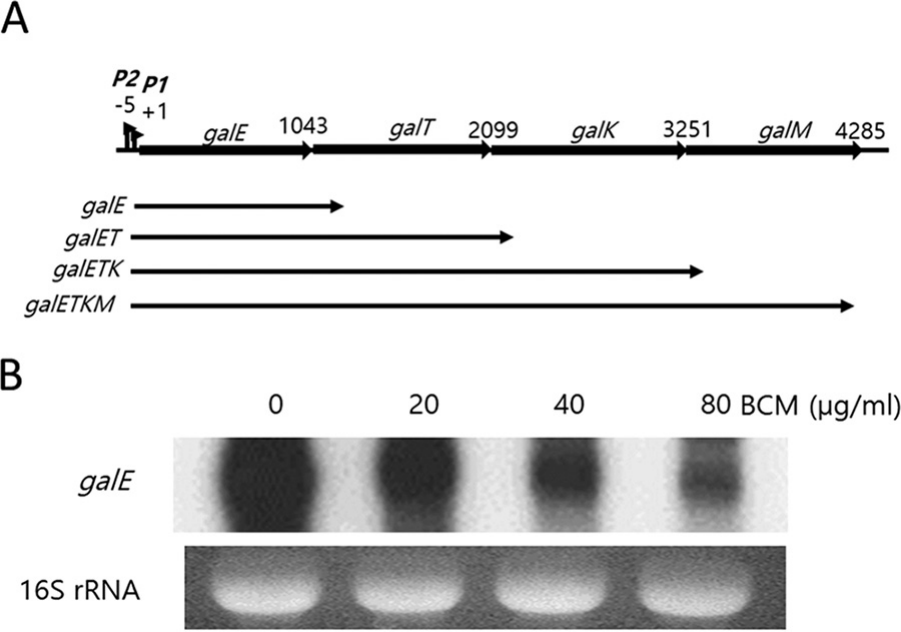
RDT is involved in the generation of *galE* mRNA. (A) The *gal* operon and the mRNA species generated from the operon. Numbers denote the last nucleotide position (from the P1 promoter transcription initiation site, +1) of the preceding gene stop codons in each cistron junction. (B) Northern blot of the *galE* mRNA. To see if the *galE* mRNA is generated by RDT, we treated MG1655 cells at an OD_600_ of 0.6 in LB medium with the indicated concentrations of the Rho inhibitor bicyclomycin (BCM) for 10 min. Total RNA from the cultures was subjected to Northern blotting.

The *gal* operon is composed of four structural genes (12, 14) and produces four mRNA species (Fig. 1A). Research suggests that the 3′ ends of the mRNA species are generated at the end of each cistron by RDT (1, 14–16). This orientation establishes polarity in gene expression (10, 14–16). At the three cistron junctions, the distance between the stop codon of the preceding cistron and the start codon of the next cistron is less than 10 nucleotides, which is insufficient for Rho to bind to the mRNA and terminate transcription if translation of the next cistron immediately follows that of the previous cistron. Therefore, in contrast to the end of the operon, where translation termination at the last stop codon 73 nucleotides upstream from the end of the full-length transcript enables RDT, it is unclear what causes RDT at the cistron junctions.

In this study, we investigated RDT at the first cistron junction of the *gal* operon, *galE–galT*, where RDT is known to generate the stable mRNA species referred to as *galE* (Fig. 1A). We found that RDT occurs near the start of *galT*, 143 nucleotides downstream from the *galE* stop codon. In addition, we investigated the effects of *galE* translation termination and *galT* translation initiation on RDT. Our results indicated that mutations that deterred *galT* translation initiation promoted RDT, whereas inhibition of *galE* translation termination inhibited RDT. We also found that a pause in the transcription of 13 nucleotides upstream from the RDT site in *galT* was necessary for RDT to occur. These results indicate that failure of *galT* translation initiation causes termination of transcription that pauses downstream of the *galT* start codon.

## RESULTS

### RDT at the *galE–galT* cistron junction generates *galE* mRNA.

RDT downstream of the first cistron junction in the *gal* operon is thought to produce the *galE* mRNA (previously known as mE1 [14]) (Fig. 1A). We, therefore, hypothesized that bicyclomycin (BCM), a Rho inhibitor, would inhibit *galE* mRNA production *in vivo*. To test this, we treated wild-type (WT) E. coli MG1655 cells with 20 μg/mL, 40 μg/mL, or 80 μg/mL (final concentration) BCM. After 10 min additional culture, we harvested the cells, extracted total RNA, and performed Northern blotting using an E-probe that hybridizes to the first half of the *galE* mRNA. The results indicated that the amount of *galE* mRNA decreased as the concentration of BCM increased (Fig. 1B). In the presence of 80 μg/mL BCM, the amount of *galE* mRNA was 40% ± 10% of that in cells without BCM treatment. Because the *galE* mRNA is about 150 bases longer than the *galE* gene (Fig. 1A), these results suggest that RDT downstream from the *galE* stop codon is involved in the production of the *galE* mRNA.

Throughout this study, we introduced nucleotide changes into intact *gal* operon DNA cloned into the single-copy plasmid pGal (15). We assayed the consequences of these mutations using E. coli MG1655Δ*gal* cells (in which the entire *gal* operon was deleted from the chromosome) harboring the pGal plasmid. We used rapid amplification of cDNA ends (3′ RACE) to target the 3′ ends of mRNA transcripts produced in the E. coli cells. By hybridizing an extension primer with the desired region of amplified cDNA and performing a primer extension reaction, we determined the number and location of the 3′ ends of the mRNA transcripts produced from the *gal* operon.

### An E-hairpin structure appears at the 3′ end of the *galE* mRNA.

**FIG 2:**
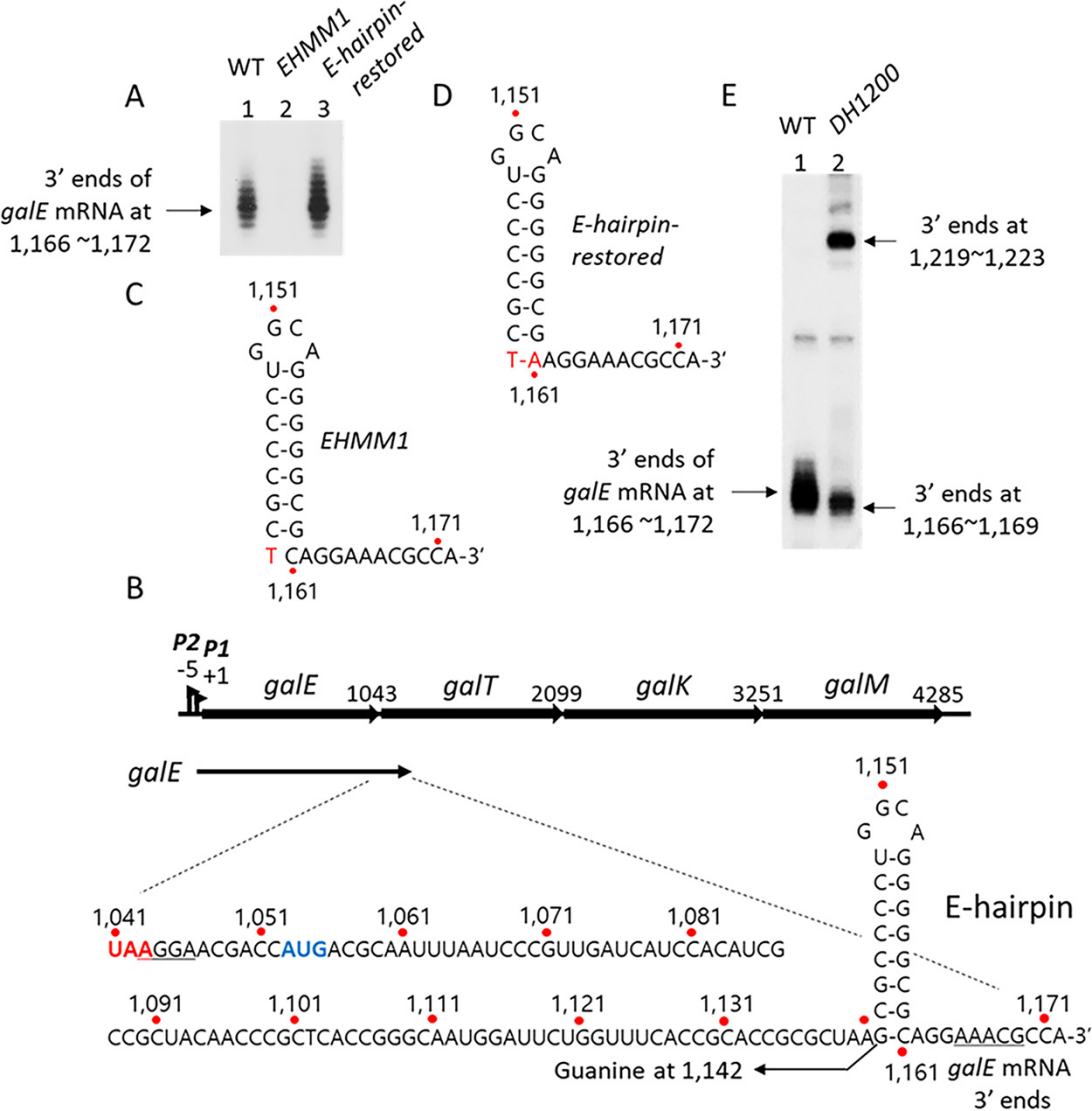
A hairpin structure, E-hairpin, at the 3′ end of the *galE* mRNA. Numbers indicate the nucleotide position from the P1 promoter transcription initiation site. (A) 3′ RACE assay of the *galE* mRNA, showing that the 3′ end formed at positions 1,166 to 1,172 in the WT (lane 1) but not in the *EHMM1* mutant (lane 2). The *E-hairpin-restored* mutant formed slightly more 3′ ends than the WT (lane 3). (B) The location and nucleotide sequence of the 3′ end of the *galE* mRNA in the WT *gal* operon. The *galE* gene stop codon is red. The *galT* gene initiator codon and SD sequence are blue and underlined, respectively. A putative hairpin structure, the E-hairpin, is drawn based on its base complementarity at positions 1,142 to 1,161. (C and D) Nucleotide changes (red) in the *EHMM1* mutant (C) and the *E-hairpin-restored* mutant (D). (E) 3′ RACE assay of *galE* mRNA in the *DH1200* mutant that has an additional E-hairpin at 1,200 (lane 2).

The 3′ RACE assay of total mRNA isolated from MG1655 cells revealed that the major 3′ end of the *galE* mRNA was located at positions 1,166 to 1,172 of the *gal* operon (Fig. 2A, lane 1), where position 1 is the transcription initiation site of the *P1 gal* promoter (Fig. 2B). These results indicated that the 3′ end of the *galE* mRNA resides about 130 nucleotides downstream from the stop codon of *galE* (Fig. 2B). Analyses of the secondary structure at the 3′ end of the *galE* mRNA revealed that a stem-loop structure of seven consecutive G·C base pairings and one G·U base pairing along with a loop of four nucleotides (called an “E-hairpin” in this study) could be formed 6 to 12 nucleotides upstream from the 3′ end of the mRNA (Fig. 2B).

In E. coli, a stem-loop structure at the 3′ end helps to stabilize mRNA by blocking 3′→5′ exoribonucleolytic digestion (1, 17, 18). To test whether the E-hairpin contributes to the stability of the *galE* mRNA, we created the *EHMM1* mutant in which the guanine (G) at position 1,142 (Fig. 2B) was changed to thymine (T) in order to abolish the bottommost 5′-G·C-3′ base pairing of the E-hairpin stem (Fig. 2C). A subsequent 3′ RACE assay indicated that the *EHMM1* mutant produced almost no *galE* mRNA 3′ ends (Fig. 2A, lane 2). To restore the bottommost base pairing of the E-hairpin, we generated the *E-hairpin-restored* mutant by changing the cytosine (C) at position 1,161 in the *EHMM1* mutant to adenine (A) (Fig. 2D). The results of a 3′ RACE assay revealed that the *E-hairpin-restored* mutant produced the same number of *galE* mRNA 3′ ends as WT cells (Fig. 2A, lane 3). These results demonstrated that the E-hairpin plays an important role in the generation of the *galE* mRNA 3′ end.

We presumed that there might be “invisible” *gal* transcripts whose 3′ ends reside somewhere downstream from the E-hairpin and that the role of the E-hairpin might be to block 3′→5′ exoribonucleolytic digestion that comes from downstream of the hairpin. To locate the 3′ ends of these invisible transcripts, we inserted a DNA segment containing the E-hairpin (42 nucleotides; 1,137 to 1,178) at position 1,200 in the WT *gal* operon to generate the double-hairpin *gal* mutant *DH1200* (see Fig. S1 in the supplemental material). Care was taken not to break the reading frame of the *galT* gene in the *DH1200* mutant. A subsequent 3′ RACE assay indicated that the *DH1200* mutant produced about half the WT number of *galE* mRNA 3′ ends. In addition, a new cluster of 3′ ends appeared at positions 1,219 to 1,223 in the *DH1200* mutant (Fig. 2E). The new cluster of 3′ ends was located 6 to 12 nucleotides downstream from the inserted E-hairpin, the same distance between the first E-hairpin and the WT *galE* mRNA 3′ end (Fig. 2B). These results suggested that in the *DH1200* mutant, there were two invisible pre-*galE* transcripts, namely, “pre-*galE1*,” whose 3′ end resided between the two E-hairpins, and “pre-*galE2*,” whose 3′ end resided somewhere downstream from the inserted E-hairpin, and that both invisible 3′ ends were processed to generate the mature *galE* mRNA 3′ end at positions 1,166 to 1,172. Thus, the mature *galE* mRNA 3′ end might be generated by blocking of 3′→5′ exoribonucleolytic digestion by the E-hairpin.

10.1128/mbio.01287-22.1 FIG S1 DNA sequences surrounding the E-hairpin and the double-hairpin mutant *DH1200*. A schematic presentation of the WT and the *DH1200* mutant. The hairpin sequences are marked in blue. Download FIG S1, PDF file, 0.08 MB. Copyright © 2022 Jeon et al. 2022 Jeon et al. https://creativecommons.org/licenses/by/4.0/ This content is distributed under the terms of the Creative Commons Attribution 4.0 International license.

### RDT occurs at position 1,183, about 10 nucleotides downstream from the *galE* mRNA 3′ end.

Close examination of the *galE* mRNA 3′ ends in the *DH1200* mutant (Fig. 2E, lane 2) suggested that the 50% decrease in 3′-end production at positions 1,166 to 1,172 occurred because the upper half of the cluster (at positions 1,170 to 1,172) was not generated. This further suggests that the invisible 3′ ends of the pre-*galE1* transcript were processed to the 3′ ends at positions 1,166 to 1,169 in the *DH1200* mutant. We hypothesized that the invisible 3′ ends of the pre-*galE1* transcript were generated by RDT.

**FIG 3:**
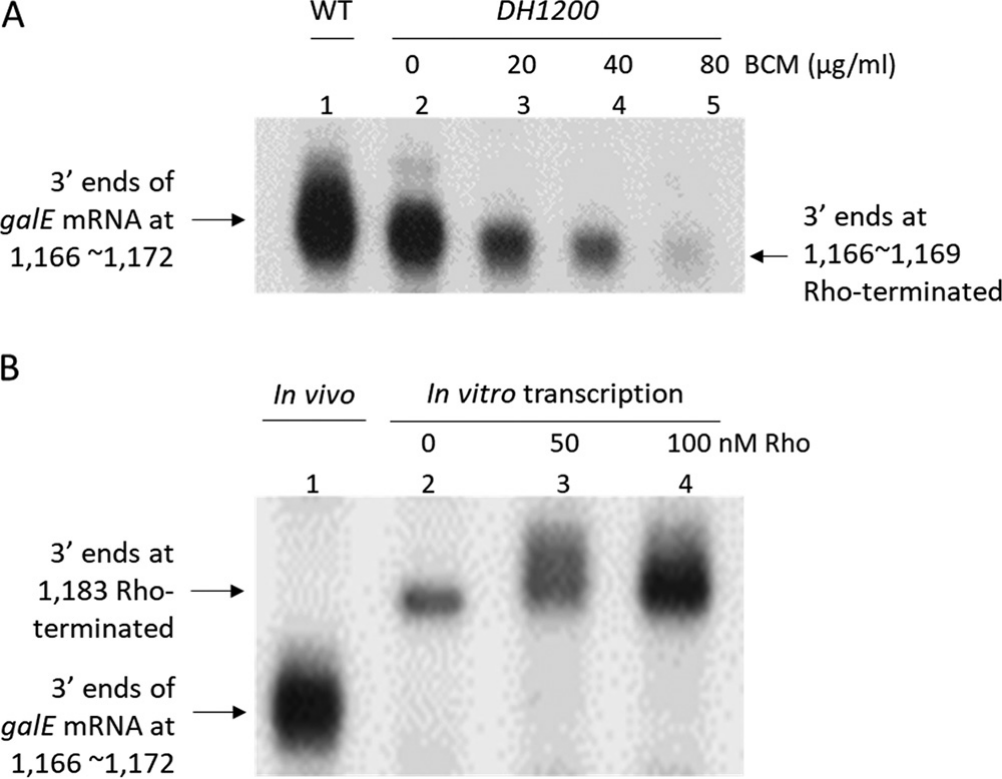
RDT at position 1,183, about 10 nucleotides downstream from the 3′ end of the *galE* mRNA. (A) 3′ RACE assay of the 3′ ends of the *galE* mRNA in the *DH1200* mutant in the presence of increasing BCM concentrations. (B) 3′ RACE assay of the 3′ ends of transcripts generated by *in vitro* transcription in the presence of the indicated concentrations of Rho protein (see the text). The 3′ end at position 1,183 in lane 2 might have been generated by Rho contamination in the RNA polymerase preparation used in the *in vitro* transcription reaction.

We reasoned that treatment of the *DH1200* mutant cells with BCM would inhibit the generation of the 3′ ends at positions 1,166 to 1,169. To test this, we treated MG1655Δ*gal* cells harboring the *DH1200* mutant operon with increasing concentrations of BCM. Subsequent 3′ RACE assays revealed that treatment with 20 μg/mL, 40 μg/mL, and 80 μg/mL BCM reduced the number of 3′ ends at positions 1,166 to 1,169 to 50%, 30%, and 10% of the WT level, respectively (Fig. 3A). These results indicate that the 3′ ends at positions 1,166 to 1,169 in the *DH1200* mutant are generated by RDT.

We performed an *in vitro* transcription reaction in the presence or absence of Rho using the plasmid pGal as a DNA template to transcribe the entire *gal* operon. We then assayed the 3′ ends of the *in vitro* transcripts that resided downstream from the E-hairpin. The results revealed that in the presence of only RNA polymerase, the 3′ ends at positions 1,166 to 1,172 were missing in the *in vitro* transcripts, although we observed 3′ ends at position 1,183 (Fig. 3B, lane 2). The number of 3′ ends at position 1,183 increased three and five times when 50 nM and 100 nM Rho were added to the transcription reaction, respectively (Fig. 3B, lanes 3 and 4) (19). These results suggest that the *in vitro* transcripts with 3′ ends at position 1,183 are generated by RDT. Taken together, the *in vitro* and *in vivo* data demonstrate that Rho terminates transcription at position 1,183 and that the resulting 3′ end is processed to generate the 3′ end of the *galE* mRNA at positions 1,166 to 1,169, thus creating the lower half of the *galE* mRNA 3′-end cluster.

### Failure of *galT* translation initiation causes RDT at position 1,183.

**FIG 4:**
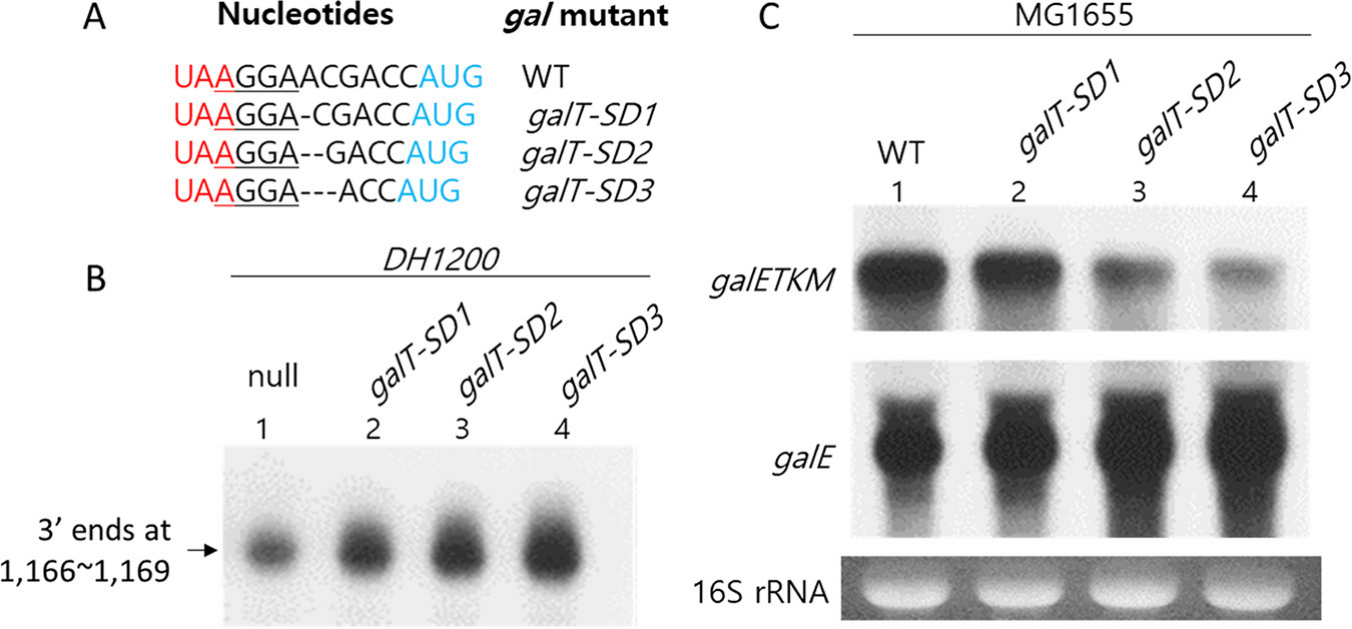
A failure of *galT* translation initiation causes RDT at position 1,183. (A) Nucleotide sequence of *gal* mutants that harbor single (*galT-SD1*), double (*galT-SD2*), or triple (*galT-SD3*) nucleotide deletions between the SD sequence and the *galT* initiator codon. The *galE* stop codon is red. The *galT* initiation codon is blue. The *galT* SD sequence is underlined. (B) 3′ RACE assay of the SD–AUG mutants in the *DH1200* background. (C) Northern blot of *galE* and *galETKM* mRNAs in the SD–AUG mutants.

The cistron junction between *galE* and *galT* is composed of just nine nucleotides (Fig. 4A). There is a putative Shine–Dalgarno (SD) sequence (5′-AGGA-3′) six nucleotides upstream of the initiator codon (AUG) of the *galT* gene. We tested the effect of *galT* translation initiation on RDT at position 1,183 by assaying the 3′ ends generated at positions 1,166 to 1,169 in the *DH1200* mutant.

We generated a series of *gal* mutants with one, two, or three nucleotides missing between the SD sequence and the initiator codon of *galT*, which we named *galT-SD1*, *galT-SD2*, and *galT-SD3*, respectively (Fig. 4A). Because these *gal* mutants had a shorter SD–AUG distance than the WT operon, we expected that the initiation of *galT* translation would become more difficult as the SD–AUG distance shortened. Contrary to our expectation, we found that the number of 3′ ends generated at positions 1,166 to 1,169 was inversely proportional to the SD–AUG distance, increasing to 120%, 180%, and 220% of the WT level in the *galT-SD1*, *galT-SD2*, and *galT-SD3* mutants, respectively (Fig. 4B). These results demonstrate that a decrease in *galT* translation initiation increases RDT at position 1,183 of the *gal* operon.

From these results, we expected a gradual increase in *galE* mRNA production with decreasing SD–AUG distance in the SD–AUG mutants. To test our hypothesis, we performed Northern blotting of the RNAs produced by the mutants and probed the blots with the E-probe. Our results confirmed that the *galT-SD1*, *galT-SD2*, and *galT-SD3* mutants produced 120%, 150%, and 200% of the WT level of *galE* mRNA, respectively; however, the amount of full-length *galETKM* mRNA was decreased to 80%, 50%, and 30% of the WT level in the same mutants, respectively (Fig. 4C). These results indicated that less transcription reached the end of the *gal* operon in the SD–AUG distance mutants than in the WT operon. Taken together, our results confirmed that increased failure of *galT* translation initiation caused an increase in RDT, suggesting that failure of *galT* translation initiation was the cause of RDT at position 1,183.

### Removal of *galE* translation termination inhibits RDT at position 1,183.

**FIG 5:**
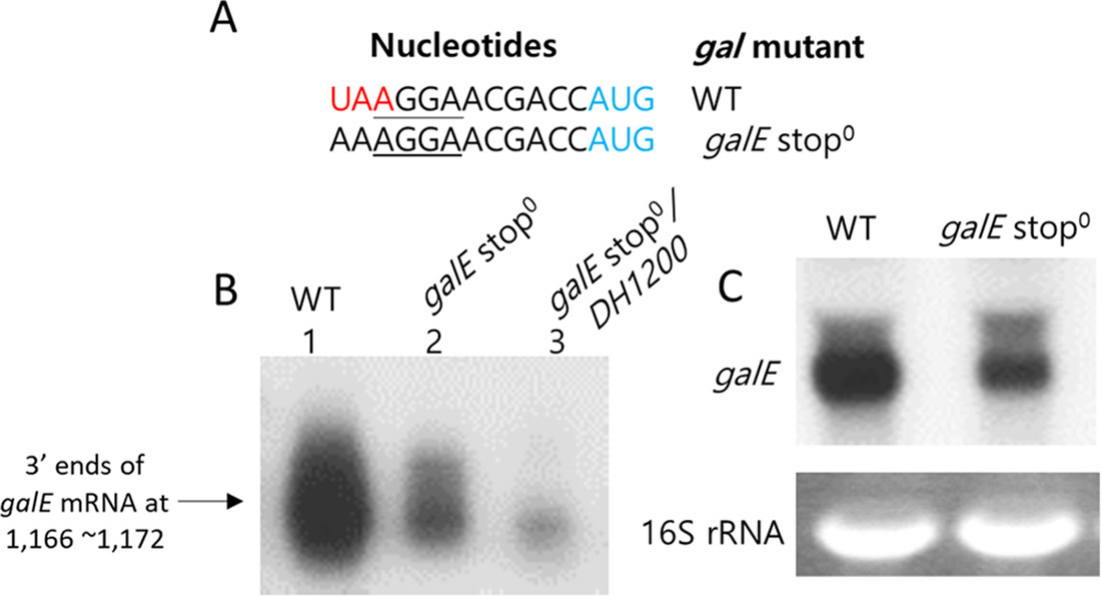
Removal of *galE* translation termination inhibits RDT at position 1,183. (A) Nucleotide sequence of the *galE* stop^0^ mutant, where the *galE* stop codon is replaced with an amino acid-coding triplet (UAA→AAA). The *galE* stop codon is red. The *galT* initiation codon is blue. The *galT* SD sequence is underlined. (B) 3′ RACE assay of *galE* mRNA in the *galE* stop^0^ mutant (lane 2) and in the double mutant *galE* stop^0^*/DH1200* (lane 3). (C) Northern blot of *galE* mRNA in the *galE* stop^0^ mutant.

To determine the effect of *galE* translation termination on RDT, we generated a *galE* stop^0^ mutant in which the stop codon of *galE* was replaced with an amino acid-coding triplet (UAA→AAA) (Fig. 5A). A 3′ RACE assay revealed that the *galE* stop^0^ mutant produced only 50% of the WT number of *galE* mRNA 3′ ends (Fig. 5B, lane 2). Furthermore, Northern blotting revealed that production of the *galE* mRNA in the *galE* stop^0^ mutant decreased to 60% of the WT level (Fig. 5C). These results showed that the prevention of *galE* translation termination had an inhibitory effect on the generation of *galE* 3′ ends, suggesting that termination of *galE* translation has a positive role in RDT.

We further reasoned that the number of 3′ ends at positions 1,169 to 1,166 in the *DH1200* mutant would decrease if we removed the termination of *galE* translation. To test this, we assayed *galE* mRNA 3′ ends in the double mutant *galE* stop^0^*/DH1200*. We generated the double mutant by inserting an E-hairpin at position 1,200 in the *galE* stop^0^ mutant. We found that the number of *galE* mRNA 3′ ends in the double mutant decreased to almost 5% of the WT number (Fig. 5B, lane 3) (20). These results confirmed that the *galE* stop^0^ mutation impaired RDT, which suggests that continuous translation at the *galE–galT* cistron junction would have an inhibitory effect on RDT.

### Transcription could pause at various sites.

**FIG 6:**
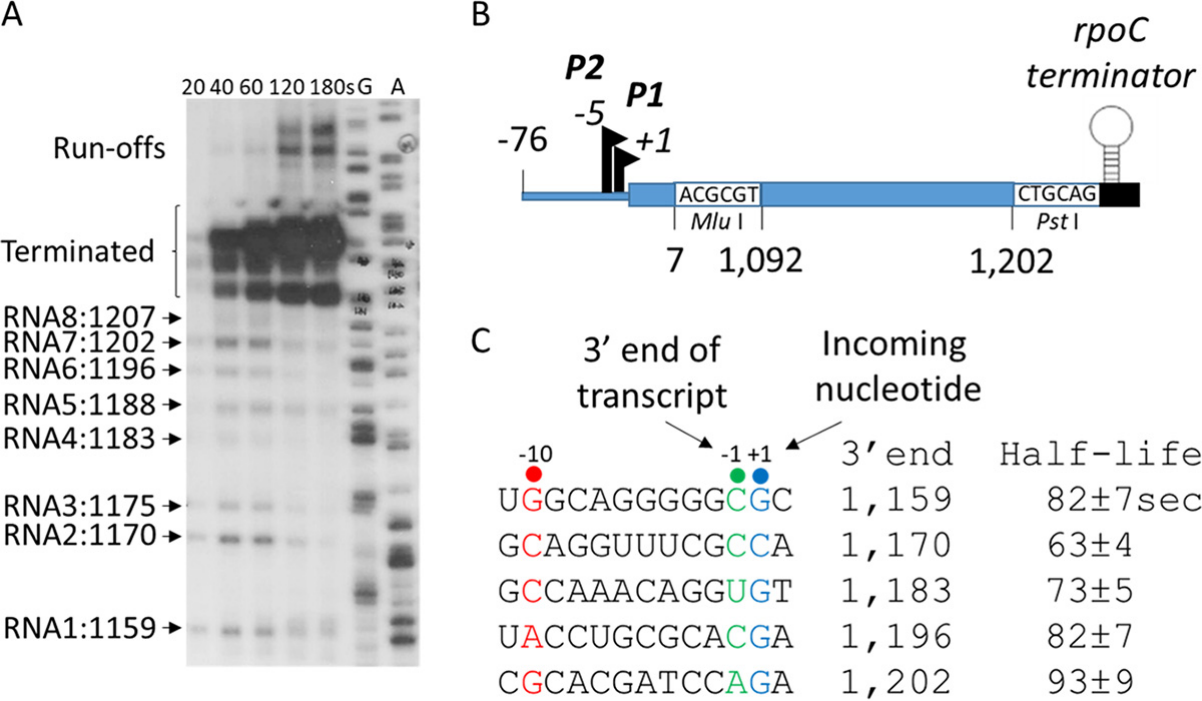
Transcription could pause at various sites. (A) RNA transcripts generated in a single-round *in vitro* transcription assay. The *in vitro* transcription reaction was performed in the presence of rifampin (100 μg/mL). Aliquots were taken out after the indicated numbers of seconds, and the transcripts generated were resolved on a DNA sequencing gel. Lanes G and A are DNA sequencing ladders. (B) The DNA template used in panel A. A *gal* DNA fragment of about 100 nucleotides that harbors the RDT site (positions 1,092 to 1,202) is cloned between the *gal* promoter region and the *rpoC* terminator using the indicated restriction sites in the plasmid pSA850, which has been used as a DNA template for *in vitro* transcription reactions (18). (C) Nucleotide sequences at the 3′ end of the RNA transcripts generated from the *P1* promoter-initiated transcription. The consensus nucleotide residues of the pausing sequences are indicated in different colors: the −10, −1, and +1 residues are indicated in red, green, and blue, respectively. The half-life of each transcriptional pause generated from the *P1* promoter-initiated transcripts is presented.

Transcription pausing by RNA polymerase is a prerequisite for transcription termination (21–23). To check for transcription pausing before RDT, we performed single-round *in vitro* transcription using a DNA template harboring the *galT* sequence from position 1,092 to position 1,202, which included the RDT site at 1,183 (Fig. S2). This DNA template (contained in the plasmid pHL3000 derived from the plasmid pSA850 [24]) was constructed so that transcription initiated from two *gal* promoters (*P1*, *P2*) could transcribe the *gal* DNA and terminate at the *rpoC* transcription terminator (Fig. 6B).

10.1128/mbio.01287-22.2 FIG S2 DNA template for the single-round of *in vitro* transcription. The consensus pause sequences are underlined showing the −10, −1, and +1 residues with reference to Fig. 6. The 3′ ends from Fig. 6 are indicated by downward arrows. The *rpoC* terminator sequences are bold and underlined. Download FIG S2, PDF file, 0.04 MB. Copyright © 2022 Jeon et al. 2022 Jeon et al. https://creativecommons.org/licenses/by/4.0/ This content is distributed under the terms of the Creative Commons Attribution 4.0 International license.

The intensity of terminated RNA bands produced in the *in vitro* transcription reaction increased with time; however, eight other RNA bands became strongest after 40 s and then decreased with time, suggesting that these RNAs were generated by transcriptional pausing (Fig. 6A). These eight RNAs had 3′ ends at sites corresponding to positions 1,159, 1,170, 1,175, 1,183, 1,188, 1,196, 1,202, and 1,207 of the *gal* operon. The *gal* coordinates start from the transcription initiation site of the *P1* promoter, so transcripts initiated from the *P2* promoter must have five more nucleotides at the 5′ end than transcripts initiated from the *P1* promoter. It is therefore likely that the RNA2 and RNA3 transcripts in Fig. 6A were initiated from the *P1* and *P2* promoters, respectively, and shared the same 3′ end at position 1,170. By the same reasoning, the RNA4 and RNA5 transcripts would share the 3′ end at position 1,183, and the RNA7 and RNA8 transcripts would share the 3′ end at position 1,202 (Fig. 6A).

The E. coli RNA polymerase often pauses transcription at G^−10^-Py^−1^-G^+1^ motifs on the template DNA strand, where Py^−1^ (cytosine or thymine) is the last nucleotide added (25, 26). Sequence analyses of the 3′ ends of the *in vitro* transcripts indicated that the nucleotides in the active site of RNA polymerases that paused at positions 1,159, 1,170, 1,183, 1,196, and 1,202 (Fig. 6C) corresponded well to G^−10^-Py^−1^-G^+1^ (25, 26). Therefore, RNA polymerase might pause transcription at these sequences *in vivo*. We measured the half-life of transcriptional pausing at the *gal* sites to be about 60 to 90 s (Fig. 6C).

### A transcriptional pause at position 1,170 (*galE* pause) is required for RDT at position 1,183 (*galT-*RDT).

Previously, we found that RDT at the end of the *gal* operon (*galM-*RDT) occurs 124 nucleotides downstream from the stop codon of *galM* (1, 14). We also found that transcription pauses 13 nucleotides upstream from the *galM-*RDT site (1). We observed the same nucleotide distance between the transcriptional pause and *galT-*RDT in this study; the transcription pause at position 1,170 (the *galE* pause) is 13 nucleotides upstream of the *galT-*RDT site. Therefore, we hypothesized that the *galE* pause is indispensable for subsequent transcription termination near the beginning of *galT*. There is support in the previous literature for this hypothesis (4, 21–23).

**FIG 7:**
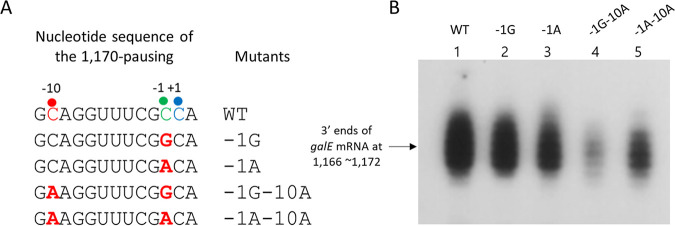
A transcriptional pause at position 1,170 is required for RDT at position 1,183. (A) Nucleotide sequence of the pause sequence at position 1,170 and nucleotide changes (red) in the pausing mutants. Changes were made in the pausing mutants −1G−10A and −1A−10A, at position 1,142 from G to T to keep the E-hairpin stem intact. The consensus nucleotide residues of the pausing sequences are indicated in different colors: the −10, −1, and +1 residues are indicated in red, green, and blue, respectively. (B) 3′ RACE assay of *galE* mRNA in the pausing mutants.

To test our hypothesis, we changed the C at the −1 position of the *galE* consensus pausing sequence to G or A, the least common nucleotides in that position. These substitutions were made in the pGal plasmid and generated the pausing mutants −*1G* and *−1A*, respectively (Fig. 7A). We then conducted a 3′ RACE assay and measured the number of *galE* mRNA 3′ ends in MG1655Δ*gal* cells harboring the pausing mutants in pGal. We found that the *−1G* and *−1A* mutants produced *galE* mRNA 3′ ends at 90% ± 7% and 70% ± 5% of the WT level, respectively (Fig. 7B, lanes 2 and 3). This suggests that the nucleotide changes at the −1 position of the *galE* consensus pausing sequence-downregulated *galT-*RDT.

In addition to the changes at the −1 position, we changed the −10C to −10A in the *−1G* mutant, generating the double-change *−1G–10A* mutant (Fig. 7A). We also made the same substitution in the *−1A* mutant, generating the *−1A–10A* mutant. It should be noted that in the two double mutants (*−1G–10A* and *−1A–10A*), we had to replace the G at position 1,142 of the *gal* operon with A so as not to disturb the E-hairpin formation. We performed a 3′ RACE assay and measured the number of *galE* mRNA 3′ ends in MG1655Δ*gal* cells harboring the double mutants in pGal. Our results indicated that the two double mutants produced *galE* mRNA 3′ ends at 50% ± 10% and 80% ± 7% of the WT level, respectively (Fig. 7B, lanes 4 and 5). The *E-hairpin-restored* mutant (Fig. 2D) with the same nucleotide changes as the double mutants at the −10 position of the *galE* pausing sequence produced *galE* mRNA 3′ ends at the WT level (Fig. 2A, lane 3). This confirmed that the decrease in *galE* mRNA 3′ ends in the double mutants (50% ± 10% and 80% ± 7% of WT in Fig. 7B) was caused by changes in the intrinsic properties of the *galE* pause, not by changes in the intrinsic properties of the E-hairpin. These results corroborated our finding that the nucleotide changes in the consensus sequence of the *galE* pause could downregulate *galT-*RDT, providing further evidence that the *galE* pause is a required step for *galT-*RDT.

## DISCUSSION

### At the cistron junction, failure to initiate translation of the next gene causes Rho-dependent termination of transcription.

We demonstrated that at the *galE–galT* cistron junction, some transcription is terminated 130 nucleotides downstream from the stop codon of *galE* by Rho, generating a stable *galE* mRNA. We measured the frequency of *galT-*RDT (Fig. S3) and found it to be 10.7%. Furthermore, we found that mutations that inhibit the initiation of *galT* translation enhance *galT-*RDT, whereas a mutation (*galE* stop^0^) that removed *galE* translation termination had the opposite effect on *galT-*RDT. These results indicated that at the *galE–galT* cistron junction, 10.7% of lead ribosomes fail to initiate translation of *galT* and thus cause *galT-*RDT.

10.1128/mbio.01287-22.3 FIG S3 Measuring the termination efficiency of the *galT-*RDT. (A) 3′ RACE assay of the 3′ end of the full-length mRNA and the *galE* mRNA generated in the MG1655Δ*gal* strain harboring the portion-deleted pGal plasmid pGalΔ1200–4200. C and A are the dideoxy DNA sequencing ladder. WT; *in vivo* 3′ RACE of the *galE* mRNA from the WT MG1655 strain. Subsequent lanes contain 1 μL, 2 μL, 3 μL, 4 μL, and 5 μL of the final 3′ RACE from the MG1655Δ*gal* strain harboring the portion-deleted pGal plasmid pGalΔ1200–4200. (B) Quantification of the mRNA 3′ ends in each lane of panel A. The basic principle for the measurement is to compare the amount of the *galE* mRNA to that of the full-length mRNA, *galETKM*. Since there are several premature RDT in the *gal* operon before the transcription termination at the end of the operon, we deleted a portion of the operon DNA from the *gal* coordinates 1,200 to 4,200, leaving the signals for the *galT*-RDT and the transcription termination at the end of the *gal* operon. We measured the 3′ ends of the *galE* mRNA and the full-length mRNA generated in MG1655Δgal strain harboring the portion-deleted pGal plasmid, pGalΔ1200–4200. To measure the radioactive signals from the RNA bands precisely, we loaded 1, 2, 3, 4, and 5 μL of the final reaction of the 3′ RACE assay. Total transcription was calculated by combining the amount from both RNA, and the efficiency of *galT*-RDT was measured as a portion of the amount of *galE* mRNA to that of the total amount in the inserted table. By taking the numbers from proportionally increasing lanes, 1, 2, and 3 μL loading, the average efficiency of the *galT*-RDT is 10.7%. These results suggested that 10.7% of transcription is terminated by Rho at 143 nucleotides downstream of the stop codon of the *galE* gene. Download FIG S3, PDF file, 0.1 MB. Copyright © 2022 Jeon et al. 2022 Jeon et al. https://creativecommons.org/licenses/by/4.0/ This content is distributed under the terms of the Creative Commons Attribution 4.0 International license.

Recently, we reported that the same principle operates at the next downstream cistron junction of the *gal* operon, *galT–galK* (27). The *galT–galK* cistron junction has three nucleotides between the stop codon of *galT* and the initiation codon of *galK*. We found that if the initiation of *galK* translation is inhibited by the binding of sRNA Spot 42, RDT occurs 84 nucleotides downstream from the *galT* stop codon, resulting in the generation of a stable *galET* mRNA (27). Thus, at the *gal* cistron junctions, and possibly at similar cistron junctions in other operons, failure in translation initiation of the next gene causes RDT near the end of the upstream gene and generates a stable mRNA species.

### At the cistron junction, failure in translation initiation of the next gene causes decoupling of translation and transcription.

In E. coli, transcription is coupled with translation (7–9). Furthermore, Rho terminates transcription when translation decouples from transcription (4, 10–13). Therefore, the fact that the *galE* stop^0^ mutation inhibited *galT-*RDT might indicate that continuous translation without termination or initiation between genes inhibits decoupling of transcription and translation. Considering that a failure of *galT* translation initiation resulted in *galE*-RDT, our results demonstrated that about 10.7% of transcription could be decoupled from the first round of translation during *galT* translation initiation at the *galE–galT* cistron junction. Contrary to the general belief that transcription–translation decoupling occurs during translation termination, we found that at the *galE–galT* cistron junction, the decoupling occurred stochastically during *galT* translation initiation.

Our results suggest that the nucleotide sequences at cistron junctions or other locations, such as promoters, might have evolved to enable stochastic failure in the initiation of translation of downstream genes. Further experiments to fully uncover the cause of the stochastic failure in *galT* translation initiation will lead to a deeper understanding of the real cause of premature transcription termination by Rho and the polarity of *gal* expression (10, 12). Previous results suggest that at the *galT–galK* cistron junction, Spot 42 binding to the beginning of the *galK* gene is the reason for the *gal* polarity (16, 27).

### A transcription model at the *galE–galT* cistron junction.

A long pause in transcription is required for RDT in the 5′-leader region of the *mgtA* gene in *Salmonella* (22). We demonstrated that transcription pauses 13 nucleotides upstream (at *gal* operon position 1,170) from the *galT-*RDT site. This pause in transcription, referred to as the *galE* pause, is an indispensable event for *galT-*RDT (Fig. 7), supporting the idea that a pause in transcription is a prerequisite for RDT (4, 21–23, 28).

Base pairing (nine nucleotides) between consensus pausing sequences of transcript RNA and template DNA in the active site of RNA polymerase makes the transcription complex go into a paused state (25, 26, 29). Structural studies of RNA polymerase assembled on RNA templates with various pausing sequences demonstrated that nucleotides upstream from the pausing sequence can form a hairpin structure in the exit channel of the paused RNA polymerase (21, 30–32). In addition, biochemical studies revealed that hairpin formation in the exit channel of the RNA polymerase could stabilize and extend the half-life of the transcriptional pause (30, 33).

**FIG 8:**
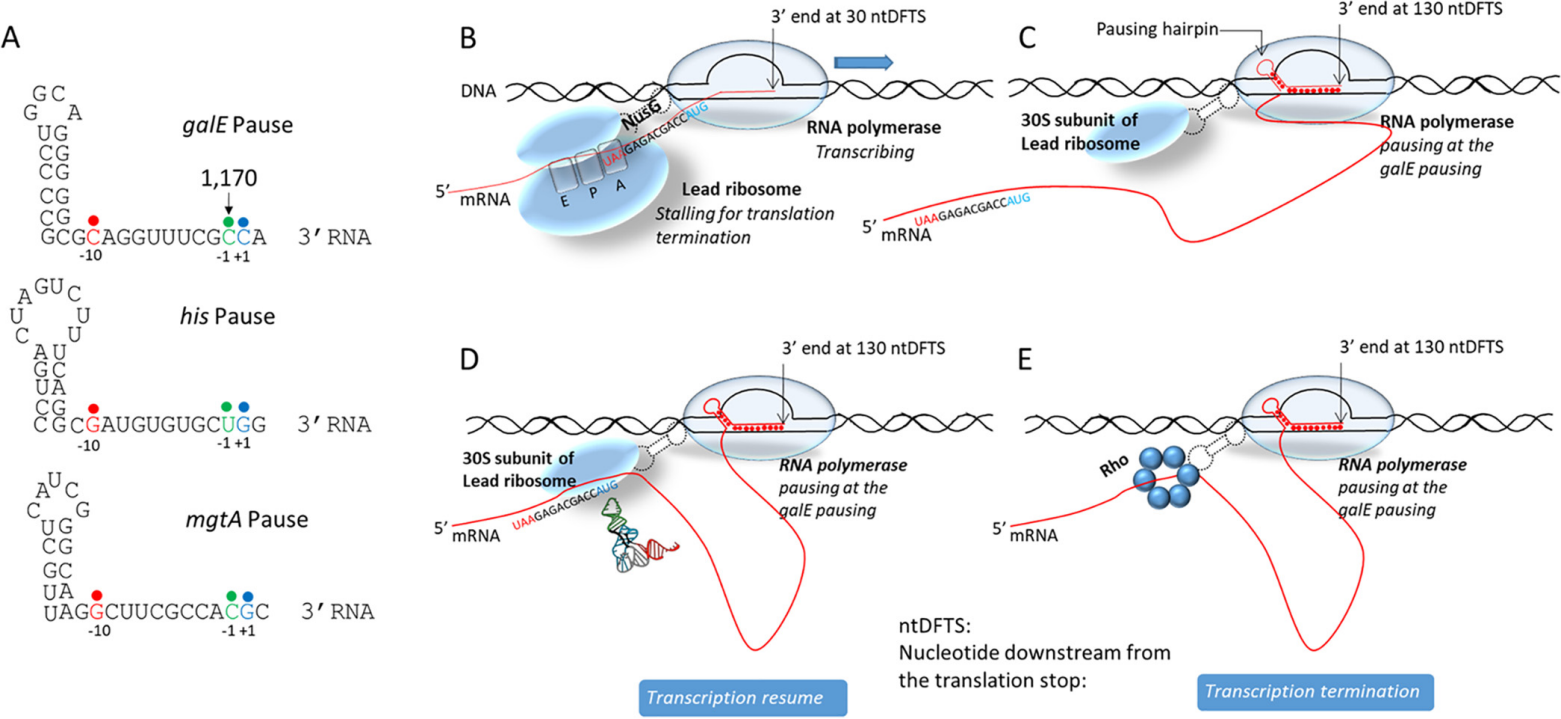
The *galE* pause sequence and transcription model at the *galE–galT* cistron junction. (A) Comparison of the G^−10^-Py^−1^-G^+1^ pausing sequences of the *galE*, *his*, and *mgtA* pauses relative to the hairpin formation. The −10, −1, and +1 residues are indicated in red, green, and blue, respectively. (B) Model of a transcription-translation complex, in which RNA polymerase is passing 30 nucleotides downstream from the *galE* gene (UAA) stop codon while the lead ribosome is terminating translation. (C) Model of a transcription-translation complex, in which RNA polymerase is pausing 130 nucleotides downstream from the *galE* stop codon while the 30S ribosome of the leading ribosome is still attached to the paused RNA polymerase after the termination of *galE* translation. Note that in this complex, the transcript RNA (red) is free from the ribosome. (D) Model of a transcription-translation complex in which RNA polymerase is paused 130 nucleotides downstream from the *galE* stop codon while the leading ribosome 30S subunit forms the preinitiation complex at the *galT* (AUG) initiation codon. A tRNA is presented over the *galT* initiation codon to emphasize the translation preinitiation complex. In these models, RNA polymerase is physically connected to the leading ribosome 30S subunit by NusG (dotted dumbbell). (E) Transcription termination model in which the NusG protein brings Rho to the RNA polymerase that is paused 130 nucleotides downstream from the *galE* stop codon.

Analyses of nucleotide sequences upstream of the pausing sequence revealed that a hairpin structure with five consecutive G·C base pairings and one G·U base pairing could form starting one nucleotide upstream from the −10 residue of the pausing sequence. This single-nucleotide distance from the stem of the pausing hairpin to the −10 residue of the pausing sequence is conserved in the well-known *his* pause (34, 35) and the *mgtA* pause (22) (Fig. 8A). Our results suggest that the *galE* pause might be another example of “hairpin-stabilized pausing,” where hairpin formation at the exit channel of RNA polymerase stabilizes the pause (25, 31, 35, 36).

In the cryo-electron microscopic structure of the ribosome and RNA polymerase complex known as the “expressome,” the distance between the active site of the RNA polymerase and the decoding center of the ribosome is about 30 nucleotides (37). Thus, when the lead ribosome arrives at the stop codon of the preceding gene, *galE*, one could imagine that the coupled RNA polymerase would be passing 30 nucleotides downstream of the stop codon (Fig. 8B) and continue transcription until it pauses transcription at the *galE* pause, 100 nucleotides further downstream (Fig. 8C).

NusG is a transcription factor that physically couples transcription to translation by connecting the ribosome to the RNA polymerase (8, 9). In the coupled transcription-translation complex, the C-terminal domain of NusG binds to the S10 ribosomal protein of the 30S ribosome subunit, and the N-terminal domain of NusG binds to the beta-subunit of RNA polymerase (8, 38–41). This physical connection between the ribosome and RNA polymerase created by NusG (8, 38–43) is represented in Fig. 8 as a dotted dumbbell shape.

When the lead ribosome terminates translation at the stop codon of *galE*, although both ribosome subunits are released from the mRNA, we propose that the 30S ribosome subunit remains connected to the paused RNA polymerase at the *galE* pause *via* NusG (Fig. 8C). This is to explain our experimental result that decoupling does not happen during *galE* translation termination. Thus, whether or not transcription resumes after the *galE* pause is influenced by the success or failure of *galT* translation initiation (Fig. 8D and E, respectively).

If the 30S ribosome subunit attached to the paused RNA polymerase binds to the SD sequence of *galT* and incorporates initiation factors and tRNA^fmet^ on the initiation codon of *galT* (Fig. 8D), with the engagement of the 50S ribosome subunit, the assembled lead ribosome initiates translation and moves on the intervening mRNA between it and the RNA polymerase, which reduces the number of nucleotides in the intervening RNA. Finally, when the size of the intervening mRNA sequence becomes 30 nucleotides, similar to the distance between the ribosome and the RNA polymerase in the expressome, we propose that the pausing hairpin is removed, releasing the RNA polymerase from the *galE* pause. From then on, transcription proceeds ahead of the lead ribosome.

NusG has been demonstrated to activate RDT (44). Its C-terminal domain has a binding affinity not only for the S10 protein of the 30S ribosome subunit but also for Rho (39, 45). Unsuccessful translation initiation at the initiation codon of *galT* causes NusG to break contact with the S10 protein and make new contact with Rho, allowing Rho to terminate transcription at the *galE* pause (Fig. 8E). We propose that the sequence for the resumption of transcription is B→C→D and the sequence for the termination of transcription is B→C→E. What causes the failure in translation initiation is unclear. It is unclear whether breaking the contact between NusG and the S10 protein is a cause or effect of decoupling. Furthermore, it is uncertain in which step the RNA polymerase transcribes the 13 nucleotides from the *galE* pause before transcription is terminated at 1,183.

### Dual roles of the E-hairpin at the 3′ end of the *galE* mRNA.

The *galT-*RDT generates a transcript RNA that has a 3′ end corresponding to position 1,183 of the *gal* operon. We refer to this transcript RNA as the invisible pre-*galE1* (Fig. 3), which becomes a *galE* mRNA after 3′→5′ exoribonuclease processing. We demonstrated that a hairpin structure at the 3′ end of the *galE* mRNA, referred to as the E-hairpin (Fig. 2), functions to block 3′→5′ exoribonuclease digestion. In the *galE* pause at position 1,170 of the *gal* operon, we propose that another E-hairpin structure with two fewer G·C base pairings at the bottom of the stem forms in the exit channel of the RNA polymerase, which we refer to as the pausing hairpin (Fig. 8A). Thus, similar stem-loop structures near the 3′ end of the mRNA can have dual roles to (i) prolong transcriptional pausing and (ii) provide transcript stability.

## MATERIALS AND METHODS

### Bacterial strains, plasmids, and growth conditions.

Chromosomal deletion strains of E. coli MG1655 were generated using the phage lambda-derived Red recombination system (46). The primers used in this study are listed in Table S1 in the supplemental material. Text S1 contains the detailed methods for plasmid construction. Prior to RNA isolation, all cells were grown at 37°C in lysogeny broth (10 g tryptone, 5 g yeast extract, and 10 g NaCl per L of water) supplemented with 0.5% (wt/vol) galactose and chloramphenicol (15 μg/mL) or ampicillin (100 μg/mL) to an optical density at 600 nm (OD_600_) of 0.6.

10.1128/mbio.01287-22.4 TABLE S1 Primers used in this study and usage. Download Table S1, PDF file, 0.1 MB. Copyright © 2022 Jeon et al. 2022 Jeon et al. https://creativecommons.org/licenses/by/4.0/ This content is distributed under the terms of the Creative Commons Attribution 4.0 International license.

10.1128/mbio.01287-22.5 TEXT S1 Plasmid construction. Download Text S1, PDF file, 0.1 MB. Copyright © 2022 Jeon et al. 2022 Jeon et al. https://creativecommons.org/licenses/by/4.0/ This content is distributed under the terms of the Creative Commons Attribution 4.0 International license.

### RNA preparation.

Total RNA was purified from clarified cell lysates using the Direct-zol RNA MiniPrep kit (Zymo Research, Irvine, CA, USA). To generate cell lysates, 2 × 10^8^ cells were resuspended in 50 μL Protoplast isolation buffer (15 mM Tris-HCl [pH 8.0], 0.45 M sucrose, and 8 mM EDTA). Five microliters of lysozyme (50 mg/mL) was added, and the sample was incubated for 5 min at 25°C. A phenolic detergent (1 mL TRI reagent; Molecular Research Center, USA) was added, and the sample was vortexed for 10 s before being incubated for 5 min at 25°C. RNA was isolated according to the manufacturer’s recommendations and dissolved in 30 μL RNA storage buffer (Thermo Fisher Scientific, USA). RNA concentrations were determined by measuring the absorbance at 260 nm using a NanoDrop spectrophotometer (Thermo Fisher Scientific, USA).

### Northern blotting.

Total RNA (10 μg in 10 μg/mL ethidium bromide) was resolved by 1.2% (wt/vol) formaldehyde-agarose gel electrophoresis at 8 V/cm for 2 h. After its integrity was evaluated under a UV light, the RNA was transferred overnight to a positively charged nylon membrane (Thermo Fisher Scientific, USA) using a downward transfer system (Whatman TurboBlotter, USA). The RNA was fixed to the nylon membrane by baking at 80°C for 1 h. Northern blot probes were prepared as follows. First, a 500-bp DNA fragment from the *galE* region (nucleotide positions +27 to +527) was prepared by PCR, followed by labeling with ^32^P. Template DNA (0.15 pmol) was mixed with random hexamers (4 μL of 1 mM) in a total volume of 28 μL and then heated at 95°C for 3 min. The reaction was then rapidly cooled for 5 min on ice, and a mixture containing 5 μL 10× Klenow fragment buffer, 5 μL deoxynucleoside triphosphate (dNTP) mix (0.2 mM dATP, dGTP, and dTTP), 10 μL α^32^P-dCTP, and 2 μL Klenow fragment (2 U/μL) was added. The reaction mixture was incubated at 37°C for 1 h, followed by inactivation of the Klenow fragment at 65°C for 5 min. The product was purified by passage through a G-50 column. The blot was prehybridized in 7 mL ULTRAhyb ultrasensitive hybridization buffer (Invitrogen, USA) at 65°C for 30 min. The DNA probe was denatured at 95°C for 5 min, and 5 μL of the probe was added to the hybridization buffer. The hybridization was performed at 42°C overnight before the blot was washed twice in low-stringency wash buffer (2× SSC [1× SSC is 0.15 M NaCl plus 0.015 M sodium citrate], 0.1% SDS) for 5 min at room temperature and then twice in high-stringency wash buffer (0.2× SSC, 0.1% SDS) for 15 min at 42°C. Finally, the radioactive bands were visualized after exposure to X-ray film. The films were scanned, and the RNA bands were quantified using the ImageJ software (NIH, USA).

### Rapid amplification of cDNA ends.

Contaminant DNA in the RNA preparation was removed using Turbo DNase I (Thermo Fisher Scientific, USA). To amplify the 3′ RNA ends (3′ RACE), we set up an RNA ligation in a 25-μL reaction volume containing 1 μL 100-nM synthetic RNA oligonucleotide (27-mer; Table S1), 2.5 μg total RNA, 2.5 μL 10× reaction buffer (Thermo Fisher Scientific, USA), 10 U T4 RNA ligase (Thermo Fisher Scientific, USA), and 10 U rRNasin RNase inhibitor (Promega). The ligation reaction mixture was incubated at 37°C for 3 h, and the resulting RNA was purified with a G-50 column (GE Healthcare, USA). The ligated RNA (4 μg) was reverse-transcribed at 37°C for 2 h in a 20-μL reaction mixture containing 4 U Omniscript reverse transcriptase (Qiagen, Germany), 0.5 mM each dNTP, 10 μM random hexamers (TaKaRa, Japan), and 10 U rRNasin. The specific 3RP primer (Table S1), which hybridizes to the ligated RNA, was used. For PCR amplification of the target RNA, 2.5 μL hybridized RNA was used as the template with a target RNA-specific primer set and HotStarTaq DNA polymerase (Qiagen, Germany) in a 50-μL reaction volume. The amplified cDNA was purified and used as a template for the primer extension reaction, which was performed in a 20-μL volume with a ^32^P-labeled specific primer and one unit of *Taq* polymerase (Qiagen, Germany) as described previously (14). The reaction products were resolved on an 8% (wt/vol) polyacrylamide–urea sequencing gel, and the radioactive bands were visualized using X-ray films. Quantification of the RNA bands was performed by scanning the film using the ImageJ software.

### *In vitro* transcription.

The pHL1277 plasmid (pGal) was used as a DNA template for *in vitro* transcription. The *in vitro* transcription reaction was performed using E. coli σ70 (Epicentre) according to the manufacturer’s protocols. Briefly, the DNA template (2 nM) was incubated at 37°C for 5 min in a reaction buffer (20 mM Tris-acetate [pH 7.8], 10 mM magnesium acetate, 200 mM potassium glutamate, 1 mM ATP, and 1 mM dithiothreitol [DTT]) containing 2 U σ70 and 40 U rRNasin (Promega) in a 47.5-μL reaction. Rho protein was added at concentrations of 50 nM and 100 nM. The reaction was initiated by adding 2.5 μL NTP mix (final concentration, 0.1 mM each NTP). After 30 min, the reaction was terminated by phenol-chloroform extraction. Then, 30 μL of the supernatant was purified using a G-50 column. The purified RNA was then utilized for 3′ RACE assays as described previously. Rho protein was purified as described previously (16). The pHL3000 plasmid was used as a DNA template for a single-round *in vitro* transcription assay. The *gal* sequences from position +1092 to position +1202 were obtained *via* PCR amplification and ligated between the MluI and PstI genes on the pSA850 plasmid (24). The single-round *in vitro* transcription reaction was performed with a DNA template (Fig. 6B), 1 U RNAP (Holo), and 40 U rRNasin (Promega) in a 36-μL reaction as described previously (47). The reaction mixture was incubated for 5 min, and 5 μL of 100-μg/mL rifampin was then added and incubated at 25°C for 1 min. The reaction was initiated at 37°C by adding 2.5 μL NTP mix (final concentration of 0.1 mM each NTP), 1 μL 5× transcription buffer, and 0.5 μL a-32P UTP to the mixture. A reaction sample (5 μL) was collected every 20 s for 120 s. The reaction was stopped by mixing with 2× stop buffer. The reaction products were resolved on an 8% (wt/vol) polyacrylamide–urea sequencing gel, and the radioactive bands were visualized using X-ray films.
