# Supplementary material for: Failure of Translation Initiation of the Next Gene Decouples Transcription at Intercistronic Sites and the Resultant mRNA Generation
Source: mBio. 2022 Jun 13;13(3):e01287-22. doi: 10.1128/mbio.01287-22 (PMC9239205; doi:10.1128/mbio.01287-22)
Supplement: FIG S1 [file mbio.01287-22-s0001.pdf]

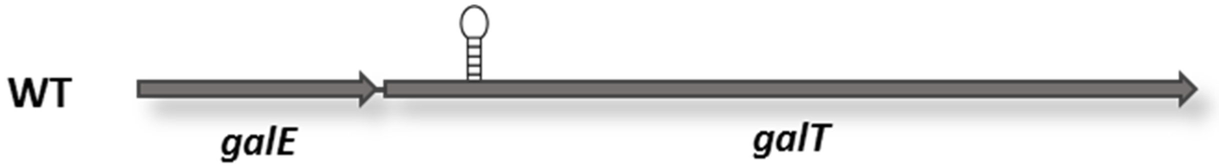

5′ 1101-CTCACCGGGCAATGGATTCTGGTTTCACCGCACCGCGCTAAGCGCCCCTGGCAGGGGGC
GCAGGAAACGCCAGCCAAACAGGTGTTACCTGCGCACGATCCAGATTGCTTCCTCTGCGCAGGTA
ATGTGCGGGTGACAGGCGATAAAAACCCCGATTACACCGGGACTTACGTTTTCACTAATGACTTTGC
GGCTTTGATGTCTGACACGCCAGATGCGCCAGAAAGTCACGATCCGCTGATGCGTTGCC-1350 3′

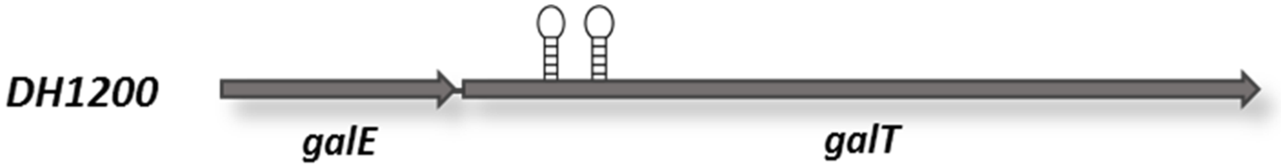

5′ 1101-CTCACCGGGCAATGGATTCTGGTTTCACCGCACCGCGCTAAGCGCCCCTGGCAGGGGGC
GCAGGAAACGCCAGCCAAACAGGTGTTACCTGCGCACGATCCAGACGTCGCTAAGCGCCCCTGGC
AGGGGGCGCAGGAAACGCCAGCCAAAGACGTCTTCCTCTGCGCAGGTAATGTGCGGGTGACAGG
CGATAAAAACCCCGATTACACCGGGACTTACGTTTTCACTAATGACTTTGCGGCTTTGAT-1300 3′
