# Supplementary material for: Failure of Translation Initiation of the Next Gene Decouples Transcription at Intercistronic Sites and the Resultant mRNA Generation
Source: mBio. 2022 Jun 13;13(3):e01287-22. doi: 10.1128/mbio.01287-22 (PMC9239205; doi:10.1128/mbio.01287-22)
Supplement: FIG S2 [file mbio.01287-22-s0002.pdf]

-76 GAATTC TTGTGTAAAC GATTCCACTA

P2

-50 ATTTATTCCA TGTCACACTT TTCGCATCTT TGTTATGCTA TGGTT**A**TTTC

P1 -10

+1 **A**TACCATACG CGTTACAACC CGCTCACCGG GCAATGGATT CTGGTTTCAC

1129 *Mlu* I 1159 1170

-1+1 -10 -1+1 -1+1 -10

+51 CGCACCGCGC TAAGCGCCCC TGGCAGGGGG CGCAGGAAAC GCCAGCCAAA

1183 1196

-1+1 -10 -1+1

+101 CAGGTGTTAC CTGCGCACGA TCCACTGCAG **CAAATAACGT AAAAACCCGC**

pst I

+151 **TTCGGCGGGT TTTTTATGG GGGG**AGTTT

rpoC terminator
