# Supplementary material for: Failure of Translation Initiation of the Next Gene Decouples Transcription at Intercistronic Sites and the Resultant mRNA Generation
Source: mBio. 2022 Jun 13;13(3):e01287-22. doi: 10.1128/mbio.01287-22 (PMC9239205; doi:10.1128/mbio.01287-22)
Supplement: FIG S3 [file mbio.01287-22-s0003.pdf]

A

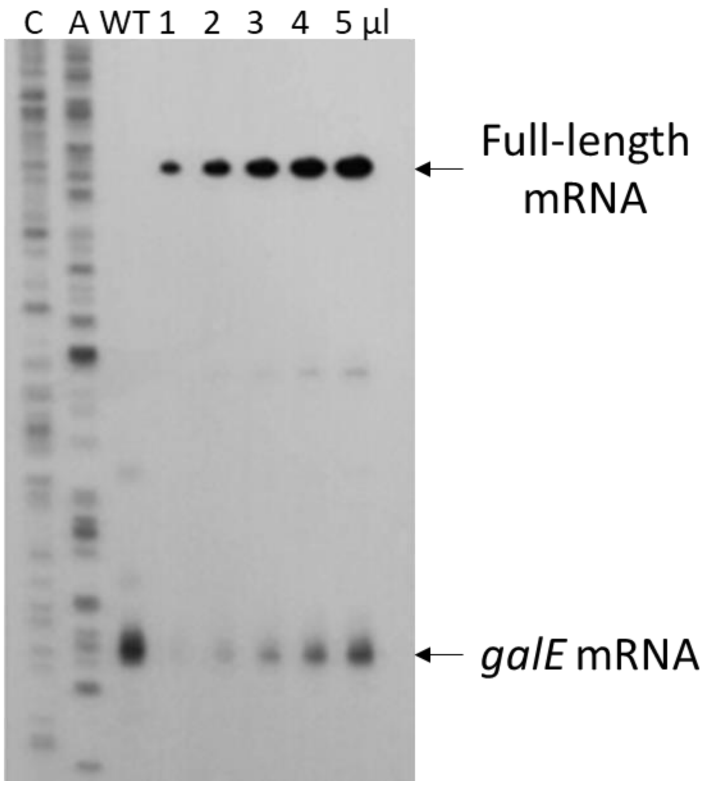

B

	1 µl	2 µl	3 µl	4 µl	5 µl
**Full-length mRNA**	6,200	12,800	18,100	22,100	26,200
***galE* mRNA**	740	1,500	2,200	3,000	3,700
**SUM**	6,920	14,300	20,300	25,100	29,700
**Termination frequency: *galE*/SUM**	10.6%	10.4%	10.8%	11.9%	8.2%
