# Supplementary material for: Failure of Translation Initiation of the Next Gene Decouples Transcription at Intercistronic Sites and the Resultant mRNA Generation
Source: mBio. 2022 Jun 13;13(3):e01287-22. doi: 10.1128/mbio.01287-22 (PMC9239205; doi:10.1128/mbio.01287-22)
Supplement: TABLE S1 [file mbio.01287-22-s0004.pdf]

Supplementary Table 1. Primers used in this study and usage.

Primer name	Primer sequence 5' to 3'	Usage
-75 *Eco*RI-F	GCGAATTCAATTCTTGTGTAAACGATTCCAC	Cloning the entire *gal* operon into pCC1BAC
*gal*BamHI-R	CCGGATCCGGTGATTTGAACAATATGAG	
*gal*HindIII-F	CACCGTTTATGGCGATCAGCCC	Cloning
*gal*MluI-R	GCGTTTTCAGTCAGTATATGACG	
EHMM1-F	TGCCAGGGGCGGTTAGCGCGGTGCGGTGA	Site-directed mutagenesis
EH-restored-R	GCGTTTCCTTCGCCCCCTGCCAGGGGCGAT TAGCGCGGT	
ENO-stop-R	GGTCGTTCCTTTATCCGGATATC	
1050_SD-1-F	GATTAAGGAACGCCATGACGCAATTT	
1050_SD-2-F	GATTAAGGAACGCATGACGCAATTT	
1050_SD-3-F	GATTAAGGAACGATGACGCAATTT	
DH1200aatII-F	GTTACCTGCGCACGATCCAGACGTC	
DH1200aatII-R	ACATTACCTGCGCAGAGGAAGACGTC	
850 MluI-R	CCATTACGCGTATGGTATGAAATAACC	
850 PstI-R	CAAATTCACGCCTGCAGGCGCCTGATT	
E1-F	ATGAGAGTTCTGGTTACCGGTGGTAG	E-probe generation - Northern blot assay
E1-R	TGGGCTTTTTTGCAGATCGGTGAGGA	
3RP	AGCATGCGGCCGCTAAGAAC	3' RACE - Reverse transcription and PCR
3’ RNA Oligo	UUCACUGUUCUUAGCGGCCGCAUGCU	
E3-F	CATCGCCCAGGTTGCTGTAG	
NewT6_1123-F	TTTCACCGCACCGCGCTAA	3' RACE - Extension primer
T1, T-ext1,T6-F	ATGACGCAATTTAATCCCGTTGATCATC	
gal+1170CtoG-R	ACACCTGTTTGGCTGCCGTTTCCTGCGCCC CCT	
gal+1170CtoA-R	ACACCTGTTTGGCTGTCGTTTCCTGCGCCC CCT	

gal+1161CtoA-R	GCGTTTCCTTCGCCCCCTGCCAGGGGCGCT	Construction of pausing mutants plasmid
gal+1161A-+1170CtoG-R	CTGTTTGGCTGCCGTTTCCTTCGCCCC CT	
gal+1161A+1170 CtoA-R	CTGTTTGGCTGTCGTTTCCTTCGCCCCCT	
