# Supplementary material for: Failure of Translation Initiation of the Next Gene Decouples Transcription at Intercistronic Sites and the Resultant mRNA Generation
Source: mBio. 2022 Jun 13;13(3):e01287-22. doi: 10.1128/mbio.01287-22 (PMC9239205; doi:10.1128/mbio.01287-22)
Supplement: TEXT S1 [file mbio.01287-22-s0005.pdf]

SUPPLEMENTARY METHOD

Plasmid construction. The pHL1277 plasmid was constructed by inserting the galactose operon (from -75 to +4333) between the *Eco*RI and *Bam*HI sites of pCC1BAC (Epicenter Biotechnologies, USA). PCR primers used to amplify the galactose operon from genomic DNA are listed in Table S1. For site-directed mutagenesis, synthetic primers containing the desired mutations were designed. After obtaining the DNA fragments containing different mutants of the *gal* operon through PCR amplification of the pHL1277 plasmid, the DNA fragments were used as a “mega primer” for the next round of PCR (1). The resulting PCR fragments were digested with *EcoR*I and *Hind*III and then ligated into pHL1277 (2) to generate pHL1750 (*EHMM1*), pHL2185 (*E-Hairpin-restored*), pHL1930 (*DH1200*), pHL2088 (*galT-SD1/ DH1200*), pHL2089 (*galT-SD2/ DH1200*), pHL2090 (*galT-SD3/ DH1200*), pHL1657 (*galE* stop° mutant) and pHL1931 (*galE* stop°*/ DH1200*). For the construction of pausing mutants -1G, -1A, -1G -10A, and -1A -10A (Fig 7B), the DNA fragments containing different mutants of the *gal* operon through PCR amplification of the pHL1277 plasmid were obtained and ligated between *EcoRI* and *HindIII* of pHL1277, generating plasmids pHL1963, pHL1965, pHL2253, and pHL2252.

REFERENCES

1. Tyagi R, Lai R, Duggleby RG. 2004. A new approach to 'megaprimer' polymerase chain reaction mutagenesis without an intermediate gel purification step. BMC Biotechnol 4:2.

2. Wang X, Ji SC, Yun SH, Jeon HJ, Kim SW, Lim HM. 2014. Expression of each cistron in the gal operon can be regulated by transcription termination and generation of a galk-specific mRNA, mK2. J Bacteriol 196:2598-606.

3. Wang X, Ji SC, Jeon HJ, Lee Y, Lim HM. 2015. Two-level inhibition of galK expression by Spot 42: Degradation of mRNA mK2 and enhanced transcription termination before the galK gene. *Proceedings of the National Academy of Sciences of the United States of America* **112**: 7581-7586.

4. Wang X, N MPA, Jeon HJ, Lee Y, He J, Adhya S, Lim HM. 2019. Processing generates 3' ends of RNA masking transcription termination events in prokaryotes. *Proceedings of the National Academy of Sciences of the United States of America*.
